# A review of the role of intermittent fasting in the management of inflammatory bowel disease

**DOI:** 10.1177/17562848231171756

**Published:** 2023-05-31

**Authors:** Celeste M. Lavallee, Andreina Bruno, Christopher Ma, Maitreyi Raman

**Affiliations:** Department of Medicine, University of Calgary, Calgary, AB, Canada; Institute of Translational Pharmacology, National Research Council of Italy (CNR), Palermo, Italy; Department of Medicine, University of Calgary, Calgary, AB, Canada; Department of Community Health Sciences, University of Calgary, Calgary, AB, Canada; Cumming School of Medicine, University of Calgary, 6D33 TRW Building, 3280 Hospital Drive NW, Calgary, AB T2N 4N1, Canada; Department of Medicine, University of Calgary, Calgary, AB, Canada; Department of Community Health Sciences, University of Calgary, Calgary, AB, Canada; Snyder Institute of Chronic Diseases, University of Calgary, Calgary, AB, Canada

**Keywords:** Crohn’s disease, inflammatory bowel disease, intermittent fasting, time-restricted feeding, ulcerative colitis

## Abstract

Intermittent fasting (IF) may be a weight management strategy for patients with inflammatory bowel disease (IBD). The aim of this short narrative review is to summarize the evidence related to IF in the management of IBD. A literature search of English publications related to IF or time-restricted feeding and IBD, Crohn’s disease, or ulcerative colitis was conducted in PubMed and Google Scholar. Four publications on studies of IF in IBD were found: three randomized controlled trials in animal models of colitis and one prospective observational study in patients with IBD. The results from animal studies suggest either moderate or no changes in weight but improvements in colitis with IF. These improvements may be mediated through changes in the gut microbiome, decreased oxidative stress and increased colonic short-chain fatty acids. The study in humans was small and uncontrolled, and it did not assess changes in weight, making it difficult to draw conclusions around the effects of IF on changes in weight or disease course. Given that preclinical evidence suggests intermittent fasting may play a beneficial role in IBD, randomized controlled trials in large patients with active disease are warranted to determine whether intermittent fasting could be an integrated therapy for patients with IBD management, either for weight or for disease management. These studies should also explore the potential mechanisms of action related to intermittent fasting.

## Introduction

Increased body mass index in patients with Crohn’s disease (CD) is associated with more severe hospitalizations, early need for surgery,^1^ higher risk for active disease, and earlier loss of response to pharmacotherapy.^2^ Although obesity was once considered uncommon in inflammatory bowel disease (IBD), its prevalence has risen in parallel with the general population. Previous estimates suggested that 20–40% and 15–40% of patients with IBD had comorbid overweight or obesity, respectively.^3^ Recently a multicenter Canadian study identified that 58% of outpatients with IBD were overweight or obese,^4^ while a meta-analysis determined that obesity is associated with an increased risk of adult-onset CD.^5^ Considering the prevalence of obesity in patients with IBD, weight management strategies specific to this population are needed. Intermittent fasting (IF), which consists of intervals of fasting and feeding, has gained popularity as a potential method for weight loss and has recently been investigated in IBD. This review summarizes the available evidence for IF in the management of IBD.

## Search methods

A search of the terms ‘intermittent fasting’ or ‘intermittent AND fasting’ or ‘time-restricted feeding’ AND ‘inflammatory bowel disease’, ‘IBD’, ‘Crohn’s disease’, ‘Crohn’s’, or ‘ulcerative colitis’ was conducted for English publications in PubMed and Google Scholar. Evidence for IF in IBD was found in three animal models of colitis^6–8^ and one prospective observational study in patients with IBD.^9^ Protocols for IF vary based on length and whether caloric restriction is required. Protocols include time-restricted feeding (TRF),^8,9^ intermittent-energy restriction (IER),^8^ alternate-day fasting (ADF),^8^ a fasting-mimicking diet (FMD)^6,7^ that allows consumption of liquids that provide some energy, and 5:2 fasting that involves fasting on two non-consecutive days per week, even though none of the studies found in this review employed the method.

## Animal studies of IF in IBD

In a randomized controlled trial (RCT) of a dextran sodium sulfate (DSS)-induced mouse model of ulcerative colitis (UC), mice were randomly assigned to two cycles of either a 2-day water-only fast separated by 12 days of feeding or a 4-day FMD separated by 10 days of feeding, or to serve as either non-fasting DSS-induced controls or non-DSS-induced (naïve) controls.^6^ Mice in the FMD group consumed 50% and 10% of their habitual energy intake on days 1 and 2–4, respectively. All mice were fed regular chow *ad libitum* between cycles to regain body weight, thus only temporary changes in body weight were noted. Colonic inflammation was scored based on abundance of lymphocytes, macrophages, and neutrophils that spanned the mucosa, *muscularis mucosae*, and submucosa of the proximal colon, using the naïve group as the control. The 4-day FMD cycles decreased colonic inflammation and inflammatory markers (CD4^+^, CD8^+^, and CD11b^+^ cells in the epithelium), improved intestinal pathology, increased stem cell abundance, and improved gut microbial dysbiosis compared to no fasting. Water-only fasting increased tissue regeneration and decreased inflammatory markers (CD11b^+^ cells in the epithelium) but did not reverse intestinal pathology compared to no fasting.

In a similar RCT, mice were randomly assigned to two cycles of FMD or to serve as non-fasting DSS-induced or non-DSS-induced controls. In this study the FMD consisted of 3 days at 30% of the energy provided to the control mice followed by 4 days of regular chow *ad libitum*.^7^ Samples were taken after the 4 days of regular chow. Similar to the previous study, only temporary changes in body weight were found after each 3-day FMD that were not retained after 4 days of feeding, but the 3-day FMD cycles improved intestinal inflammation and pathology, increased colon length, and increased colonic crypt and stem cell abundance compared to *ad libitum* intake in DSS-induced mice.

Another RCT explored effects of ADF, TRF, and IER on colitis-related outcomes in preclinical models.^8,10^ A total of eight groups of mice were studied: mice fed *ad libitum* served as non-DSS and DSS controls; non-DSS mice that underwent ADF, TRF, or IER; and DSS-induced mice that underwent ADF, TRF, or IER. The fasting protocols lasted 36 days, and were as follows: ADF consisted of a 24-h fast followed by *ad libitum* chow for 24 h; TRF consisted of a 16-h fast followed by *ad libitum* chow for 8 h; IER consisted of two cycles of 4 days (days 11–14 and days 29–32) of an energy-restricted diet that provided 50% and 10% of normal energy intake on days 1 and 2–4, respectively, with *ad libitum* chow on non-energy-restricted days. Intestinal barrier integrity was assessed by transmission electron microscopy to detect the ultrastructure of colonic tissue, via mRNA expression of tight junction proteins claudin-1, zonula occludens-1 and occludin, by penetration of liposaccharide into serum from the gut determined using an enzyme-linked immunosorbent assay, and via immunohistochemical analysis of claudin-1 in colonic tissue. Colonic inflammation was assessed by histology and via mRNA expression of tumor necrosis factor-α, interleukin-1β, interleukin 6, and interferon-g. Compared to DSS-induced mice fed *ad libitum*, survival time, integrity of the intestinal barrier (all assessments showed improvement), and production of colonic short-chain fatty acids were improved in DSS-induced mice who underwent TRF and IER, but not ADF. Also compared to DSS-induced mice fed *ad libitum*, DSS-mediated shortening of the colon improved while colonic oxidative stress, assessed by the biomarker malondialdehyde, and inflammation (all assessments showed improvement) were reduced with TRF and IER. Additionally, TRF and IER improved microbial dysbiosis, demonstrated by increased abundance of Christensenellaceae, Rikenellaceae, *Lactobacillus, Coprococcus*, and *Ruminococcus*, and decreased abundance of *Shigella* and *Escherichia coli*. ADF and IER reduced body weight in control mice while DSS treatment reduced body weight in all mice; only DSS + ADF reduced body weight significantly more than in non-fasting DSS-induced mice.

## Human studies of IF in IBD

Although there have not been any RCTs investigating the effects of IF in human subjects with IBD, one prospective observational study reported the fasting experience in people with IBD during Ramadan. Negm *et al.*^9^ recruited 80 patients with either CD or UC from two IBD referral centers in Egypt. Patients were eligible regardless of their disease activity but excluded if their medication regime had changed in the previous 12 weeks or was expected to change in the upcoming month. The fasting interval for Ramadan started at roughly 14 h and increased to 15 h by the end of the month. The study assessed changes in symptoms [Harvey Bradshaw Index (HBI) for patients with CD or partial Mayo score (PMS) for patients with UC], systemic and gut inflammation [C-reactive protein (CRP) and fecal calprotectin (FCAL), respectively], quality of life, and levels of depression but did not assess changes in weight. Eighty eligible patients entered the study (20 with CD and 60 with UC). One patient was later excluded due to an iatrogenic perforation and two patients stopped fasting due to deterioration of their condition. Results from the latter were included to the days they stopped fasting (days 20 and 22). At baseline HBI ranged 0–15 [median 4, interquartile range (IQR) = 3–5] and PMS ranged 0–6 (median 1, IQR = 0–3). In patients who had CRP and FCAL measured at baseline and post-fasting, baseline median CRP (*n* = 65) was 0.53 mg/dL (IQR = 0.18–1.56 mg/dL) and baseline median FCAL (*n* = 51) was 163 µg/g (IQR = 35–418 µg/g). No differences were found in any measure except for a statistically significant increase in median PMS (post-fast median = 1, IQR = 0–5; *p* = 0.02 compared to baseline). Subgroup analysis and multiple linear regression determined the increase in PMS was associated with older age (⩾30) and higher baseline FCAL (⩾200 mg). Although median HBI increased to 5 post-fasting (IQR = 2–7), the change was not statistically significant.

## Discussion

Collectively, IF interventions in animal studies suggest improvement of colitis, possibly mediated through amelioration of gut microbial dysbiosis, decreased oxidative stress and enhanced colonic short-chain fatty acids achieved through either modest or no weight loss. While DSS-induced mouse models of colitis are commonly used, there are a number of factors that influence the effectiveness of the induction. These include batch differences in DSS, dose and duration of exposure, the strain and sex of the animal, and the microbial environment,^11^ all of which can lead to variations in disease severity^11^ and, potentially, response to therapy such as IF. As with any animal model of disease, results must be interpreted with caution. However, it is plausible that IBD could respond similarly to IF as do other immune-mediated diseases, such as psoriatic or rheumatoid arthritis. Studies in humans of the effects of IF during Ramadan on these rheumatic diseases reported improvements in disease activity and either a decrease or no change in inflammatory makers.^12,13^ Together, the evidence from other immune-mediated diseases and from these preclinical studies in IBD suggests IF may play a beneficial role in IBD, supporting the need for interventional studies in humans with CD or UC.

Although one observational study found no improvement in, albeit no worsening of, inflammation in patients with CD or UC, the study was small and non-controlled, and based on objective biomarkers included largely patients in remission or with mild disease. While the subgroup analyses identified statistically significant worsening of PMS in patients with UC who were older or had higher baseline FCAL, inflammatory markers (CRP and FCAL) were not significantly different after fasting. Given that patients were not stratified *a priori* based on disease or symptom severity, it is difficult to decipher whether patients in clinical and/or biomarker remission at baseline fared different from patients with severe disease and/or symptoms. Furthermore, since dietary intake between fasting periods was neither restricted nor assessed, and compliance with fasting was not reported, it is not known whether dietary intake or adherence to fasting differed among participants and thus could have contributed to worsening PMS in some participants. Finally, the authors of that study eloquently described a number of other limitations to the study, including that IF over 1 month during Ramadan may not be long enough to notice significant improvements or deteriorations in disease course, compliance with medications may be compromised due to restrictions to when they can be taken during Ramadan, and that comorbidities tend to be more common in older patients and these may have contributed to changes in the physician’s assessment portion of the PMS.^9^ Reasons for why these results differ somewhat from those in the studies of rheumatic diseases^12,13^ are speculative in the absence of other published literature but could be related to effects on the gut microbiome, autophagy, and other similar pathophysiologic mechanisms that underpin IBD and other immune-mediated diseases.

While preclinical evidence suggests that a beneficial effect may be conferred with IF in IBD, one should remain cautious that people with IBD may respond differently to IF than people with other immune-mediated diseases. Given the limited evidence for fasting in people with IBD it is difficult to draw conclusions regarding the effects of IF on body weight, inflammation, or disease activity. Thus, large RCTs in patients with active disease, based on objective markers of disease activity and symptoms, are warranted to better understand whether IF could be an effective and safe therapeutic option to integrate into IBD management. Such studies would also provide evidence on whether humans with IBD respond similarly to IF as do preclinical models of colitis, and thus, whether these preclinical models could be used for future studies to explore the mechanisms of action that have been evidenced in other conditions, including mechanisms related to IF and autophagy,^14,15^ circadian rhythm,^16^ white adipose tissue-browning,^17^ adipokines,^18,19^ and the adipose-tissue-gut microbiome axis.^20,21^

## Declarations

**Ethics approval and consent to participate:** Not applicable.

**Consent for publication:** Not applicable.

**Availability of data and materials:** Not applicable.
